# Novel Missense Variant in Heterozygous State in the *BRPF1* Gene Leading to Intellectual Developmental Disorder With Dysmorphic Facies and Ptosis

**DOI:** 10.3389/fgene.2020.00368

**Published:** 2020-05-07

**Authors:** Muhammad Imran Naseer, Angham Abdulrahman Abdulkareem, Francisco J. Guzmán-Vega, Stefan T. Arold, Peter Natesan Pushparaj, Adeel G. Chaudhary, Mohammad H. AlQahtani

**Affiliations:** ^1^Center of Excellence in Genomic Medicine Research, King Abdulaziz University, Jeddah, Saudi Arabia; ^2^Department of Medical Laboratory Technology, Faculty of Applied Medical Sciences, King Abdulaziz University, Jeddah, Saudi Arabia; ^3^Division of Biological and Environmental Sciences and Engineering (BESE), Computational Bioscience Research Center (CBRC), King Abdullah University of Science and Technology (KAUST), Thuwal, Saudi Arabia; ^4^Centre de Biochimie Structurale, CNRS, INSERM, Université de Montpellier, Montpellier, France; ^5^Center for Innovation in Personalized Medicine, King Abdulaziz University, Jeddah, Saudi Arabia

**Keywords:** *BRPF1*, dysmorphic facies, intellectual developmental disorder, ptosis, Saudi family

## Abstract

Intellectual developmental disorder with dysmorphic facies and ptosis is an autosomal dominant condition characterized by delayed psychomotor development, intellectual disability, delayed speech, and dysmorphic facial features, mostly ptosis. Heterozygous mutations in bromodomain and plant homeodomain (PHD) finger containing one (*BRPF1)* gene have been reported. In this study, whole exome sequencing (WES) was performed as a molecular diagnostic test. Bioinformatics of WES data and candidate gene prioritization identified a novel variant in heterozygous state in the exon 3 of *BRPF1* gene (ENST383829: c.1054G > C and p.Val352Leu). Autosomal dominant inheritance in the family affected individuals and exclusion of non-pathogenicity in the ethnically matched healthy controls (*n* = 100) were performed by Sanger sequencing. To the best of our knowledge, this is the first evidence of *BRPF1* variant in a Saudi family. Whole exome sequencing analysis has been proven as a valuable tool in the molecular diagnostics. Our findings further expand the role of WES in efficient disease diagnosis in Arab families and explained that the mutation in *BRPF1* gene plays an important role for the development of IDDFP syndrome.

## Introduction

Intellectual developmental disorder with dysmorphic facies and ptosis (IDDDFP) is an autosomal dominant neurodevelopmental disorder that can be explained as intellectual disability and dysmorphic facial features and delayed psychomotor and language development (Deciphering Developmental Disorders, 2017). Moreover, ophthalmological anomalies such as ptosis and blepharophimosis syndrome, amblyopia, strabism, and refraction problems are also included in this disorder (Mattioli et al., 2017; Deciphering Developmental Disorders, 2017; Yan et al., 2017). Some the patients also showed seizure, hypotonia, short stature, and additional features, along with microcephaly (Mattioli et al., 2017; Yan et al., 2017). Recently, another study showed that novel *de novo* nonsense mutation in *BRPF1* gene leads to intellectual disability, coloboma, facial nerve palsy, and hypoplasia of the corpus callosum (Demeulenaere et al., 2019). Mutations in *BRPF1* have also been shown to cause intellectual developmental disorder with dysmorphic facies and ptosis (MIM: 617333), an autosomal dominant condition (Mattioli et al., 2017; Yan et al., 2017). Furthermore, some of the cases with the mutation in *BRPF1* gene also leads to cause variable degrees of intellectual disability, distinct facial features including downslanted palpebral fissures, ptosis, and/or blepharophimosis (Pode-Shakked et al., 2019).

The *BRPF1* gene translates a bromodomain, plant homeodomain (PHD) finger, and chromo/Tudor-related Pro-Trp-Trp-Pro (PWWP) protein Vezzoli et al. (2010). The protein encoded by *BRPF1* gene is an important element of the MOZ/MORF histone acetyltransferase (HAT), which functions as a transcriptional regulator Ullah et al. (2008). The MOZ/MORF complex represents an example of a chromatin-binding assembly that plays an important role to fine-tune the post-translational modifications of histones. BRPF1 binds to the catalytic MYST domains of the MOZ and MORF proteins and may play a role to stimulate acetyltransferase to control the transcriptional activity of the complex (provided by RefSeq, January 2016).

Here, we report a Saudi family with novel missense variant in heterozygous state in the *BRPF1* gene leading to the intellectual developmental disorder with dysmorphic facies and ptosis.

## Results

### Clinical Report of the Patient

**FIGURE 1:**
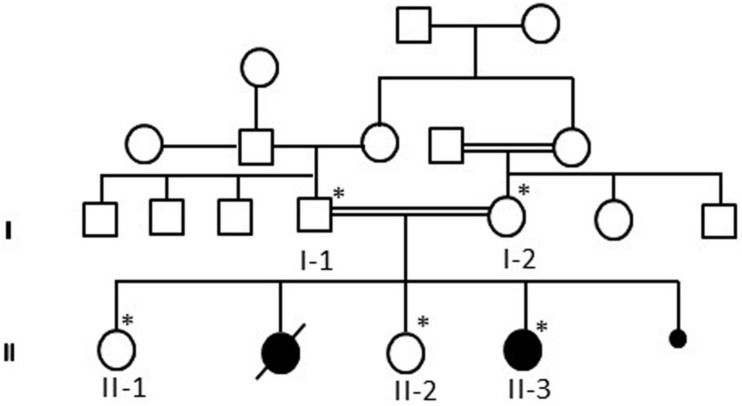
A pedigree of a consanguineous Saudi family drawn after having the details from the parents. The available samples are marked as ^∗^ satiric symbol.

**FIGURE 2:**
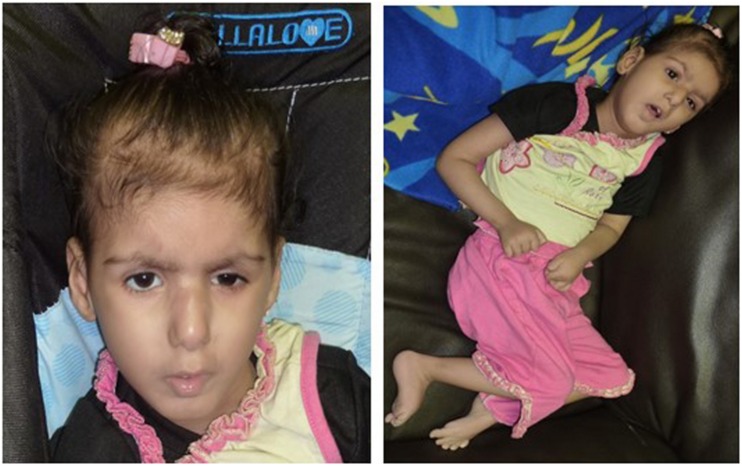
Showing the facial appearance at the age of 5 years, face with interior hairlines, narrow palpebral fissures, epicanthic folds and hypertelorism, broad eyebrows, and wide nasal bridge with prominent nasal tip along with prominent ear crus.

Patient II-3 is 6 years old, and she was born by normal vaginal delivery at full term without any complication. She has two normal sisters, and one sister died after 1 day of her birth due to the abnormal growth conditions. After 5 months of birth index, patient II-3 was admitted in the hospital as the family history was remarkable. Detailed family pedigree was drawn after having information from the family as shown in Figure 1. Earlier she was diagnosed as having microcephaly head circumference of 38.5 cm (SD, −2.8), dysmorphic features, and brain atrophy. At the age of 6 years, her face showed interior hairlines, narrow palpebral fissures, epicanthic folds and hypertelorism, broad eyebrows, and wide nasal bridge with prominent nasal tip, along with prominent ear crus (Figure 2). She is unable to walk and speak. She had hypotonia and gross motor delay, along with intellectual disability. She was also reported difficulties in swallowing, whereas no seizure was reported.

### Whole Exome Sequencing

The resulting variant call format (vcf) file with 111,850 variants was identified. These variants are filtered based on quality, genomic position, frequency, protein effect, pathogenicity, and previous associations with the phenotype. We failed to detect homozygous gene variants in the analysis, and hence heterozygous gene variants were subsequently considered. We identified a novel heterozygous missense variant in the *BRPF1* gene (c.1054G > C; p.Val352Leu). Whole exome sequencing (WES) analysis showed a pathogenic mutation in the *BRPF1* gene where G at position 1054 is replaced by C in the third exon of the gene. Thus, WES results revealed that the *BRPF1* gene showed a novel heterozygous mutation in affected member of the family. The detected variant causes the substitution of a valine into a leucine residue at position 352 in the C2H2 Zn-finger domain. This variant is absent in gnomAD and dbSNP and has not previously been associated with disease. Multiple *in silico* tools predict a deleterious effect of this missense change. The heterozygous state of this variant indicates that it is unlikely to be responsible for the shared features of all affected siblings. Furthermore, the *de novo* occurrence of this variant is also possible.

### Sanger Sequencing

**FIGURE 3:**
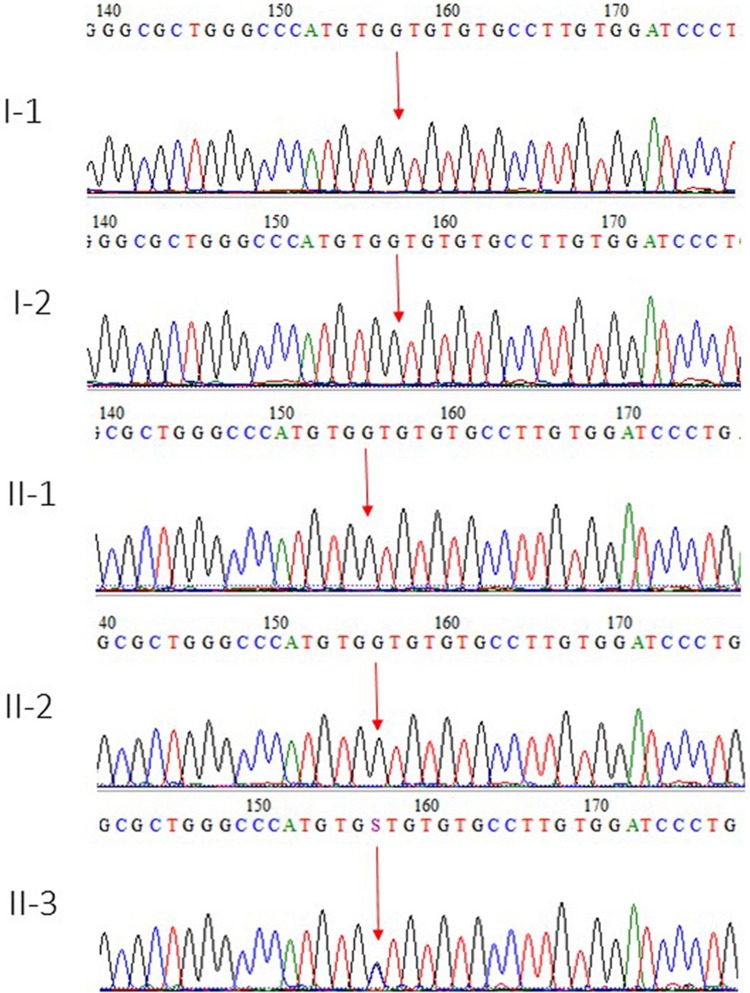
Sanger sequence analysis chromatogram showed I-1 and I-2 are normal parents, whereas II-1 and II-2 are normal siblings, and II-3 is the affected member of the family showing a novel missense variant in heterozygous state where c.1054G > C and p.Val352Leu in the exon 3 of BRPF1 gene.

Whole exome sequencing results were validated by using Sanger sequencing technique by designing the primers of the region. This mutation was also not identified in 100 unrelated healthy persons in the population. The parents and the two sisters of the affected member were also normal, whereas the heterozygous mutation was detected only in the index patient II-3, as shown in Figure 3.

### Predicted Structural Effect of BRPF1 Mutation

**FIGURE 4:**
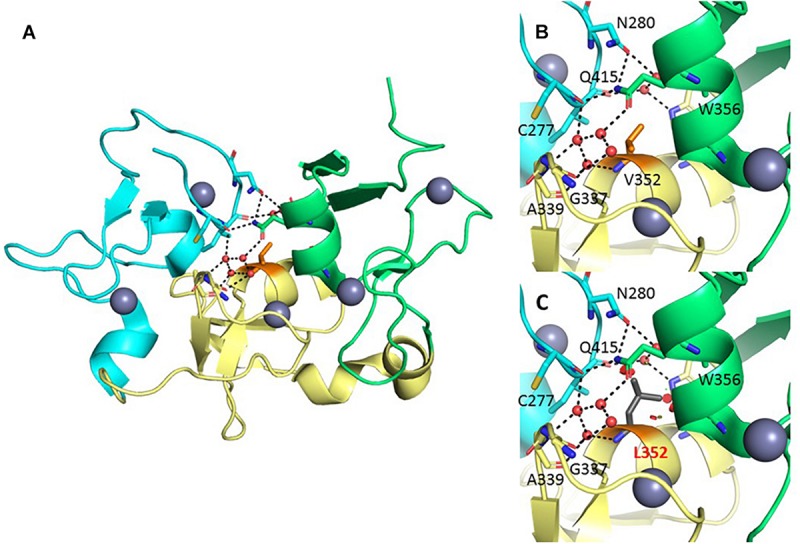
Structural analysis of the Val352Leu variant. **(A)** Crystal structure of the BRPF1 PZP domain (PDB 5ERC). The PHD1, Zn-kn, and PHD2 domains are colored cyan, yellow, and green, respectively. Zinc ions and water molecules are presented as gray and red spheres, and hydrogen bonds as black dashed lines. Val352 is shown in the center of the molecule (orange stick representation). Important residues (stick representation) and water molecules (red spheres) in the interface between the three domains are displayed. **(B)** Zoom into the region harboring Val352, in its position buried beneath the hydrogen-bond network of nearby residues and water molecules. **(C)** The substitution for the bulkier leucine would potentially destabilize the polar interactions in this region, affecting the arrangement of the PHD1, Zn-kn, and PHD2 domains, and disturbing the structural frame coordinating the three zinc ions nearby.

Val352 is located in the central region of BRPF1, in a so-called zinc knuckle (Zn-kn) domain. The Zn-kn tightly links two PHDs into a conserved PHD1–Zn-kn–PHD2 module, termed the PZP domain (Figure 4A). The isolated PHD1 finger binds to the unmodified histone H3 tail, and the PHD2 finger binds to DNA in a non-specific fashion (Qin et al., 2011; Liu et al., 2012; Klein et al., 2016). The three domains of the PZP module are joined through a zipper-like network of hydrogen bonds and hydrophobic interactions, with the Zn-kn acting as a platform to orient and keep together the PHD1 and PHD2 domains (Figure 4B). The substitution of Val352 for a leucine, a bulkier hydrophobic residue, would introduce steric clashes mainly with the neighboring residues sustaining the polar interactions that keep the PZP domain together (Figure 4C). Hence, this mutation is predicted to affect the integrity of the PZP domain. Furthermore, this substitution could compromise the stability of the three adjacent zinc-binding motifs, potentially having a negative effect on the histone and DNA-binding properties of their corresponding regions.

## Discussion

The *BRPF1* gene code is a protein that is expressed ubiquitously and at the highest expression level in testes and spermatogonia. The localization of this protein was observed within the nuclei and showed similarities with AF10 and AF17 two zinc finger proteins. Further, it is proposed that these proteins form a family of regulatory proteins (provided by RefSeq, January 2016). *BRPF1* is a core subunit of the MOZ HAT that plays an important role for normal developmental process and associated with acute leukemia. *BRPF1* contains the MOZ-binding domain in the N-terminus (Ullah et al., 2008). *BRPF1* functions as a scaffolding subunit, linking MOZ, ING5, and hEAF6 together into a functional complex (Klein et al., 2016). In this study, we report a novel variant lying in the Zn-kn fragment of the central PZP module of *BRPF1* gene. Through the resulting steric hindrance, this mutation is predicted to destabilize the PZP arrangement and hence to affect recognition of histone tails and DNA. Thus, the mutation may alter the epigenetic and developmental programs depending on such interactions with the chromatin.

*In vivo* studies demonstrated that *BRPF1* gene plays an important role in development of forebrain and acts as an activator and silencer of gene expression and decreases expression of the chromatin regulator leading to abnormal brain development (You et al., 2015a). In a mouse model study, *BRPF1* gene is expressed in the cerebellum and forebrain and plays an important role for embryogenesis and for the development of the neocortex and corpus callosum (You et al., 2015a, b). Furthermore, the Medaka fish and mouse model studies explained the role of *BRPF1* gene in skeletal and craniofacial development (Hibiya et al., 2009). *BRPF1* controls development of the forebrain while its inactivation leads to early postnatal lethality and severe growth retardation, along with aberrant behaviors (You et al., 2015c).

Moreover, *BRPF1* mutations were associated with IDDDFP (OMIM 617333) (Mattioli et al., 2017; Yan et al., 2017). So far, clinical reports have been published for 23 patients from 18 different families. The reported mutation spectrum showed frameshift, nonsense, and small deletions, along with two missense mutations. However, four cases showed the loss-of-function mutations were defined in the Deciphering Developmental Disorders study without any phenotypic descriptions (Deciphering Developmental Disorders, 2017).

A novel variant in a boy with intellectual disability, facial nerve palsy, and coloboma, along with hypoplasia of the corpus callosum, was recently reported by Demeulenaere et al. (2019). A study of 10 patients (of which nine came from unrelated families) with intellectual developmental disorder, dysmorphic faces, and ptosis found these were due to heterozygous mutations in the *BRPF1* gene (Yan et al., 2017). One mutation was a missense mutation, and all the others were nonsense or frameshift mutations leading to C-terminal truncations of the protein that differed in which structural domains were deleted. Functional studies showed that the reported BRPF1 variants were pathogenic and impaired acetylation of histone H3 at lysine 23, although they acted through different mechanisms. Mattioli et al. (2017) described that 2bp deletion resulting in a frameshift mutation in *BRPF1* in five members of a large family affected by a mild form of intellectual developmental disorder with dysmorphic faces and ptosis. In one male patient with intellectual developmental disorder with dysmorphic faces and ptosis, a heterozygous missense mutation was identified resulting in a P370S substitution, which was inherited from the patient’s unaffected mother who was mosaic for the mutation (Yan et al., 2017). The reported clinical phenotype of this patient overlaps with the manifestations of this condition regarding abnormal facial shape and microcephaly. Brain atrophy, which is also reported for this patient, is not typically associated with this condition. However, Mattioli et al. (2017) reported agenesis of corpus callosum in one patient carrying a *BRPF1* mutation [C > G (p. Tyr994^∗^)] and enlarged perivascular Virchow–Robin spaces in another patient carrying a *BRPF1* mutation (p.Val351Glyfs^∗^8). The typical age at onset of intellectual developmental disorder with dysmorphic faces and ptosis ranges from 0 to 1 years, which is in line with the reported age at onset in this patient (0 years).

Some knockout studies in mouse showed that *Brpf1* is critical for developmental delay, intellectual disability, and language impairment (You et al., 2015a,b,c). Overall, *BRPF1* gene is very important and plays a role in the development of the neocortex and corpus callosum by controlling neurogenesis and transcriptional programs.

## Conclusion

We described a patient with a novel missense variant in the *BRPF1* gene for the first time in a Saudi family. The disease phenotype of this missense *de novo* mutation in *BRPF1* with ID and dysmorphism is overlapping with the IDDDFP syndrome. Furthermore, the finding of the mutation emphasizes the important role of *BRPF1* gene in the brain and intellectual developmental disorder in humans.

## Materials and Methods

### Ethical Approval and Sample Collection

The blood samples from the whole family members were collected according to the ethical protocols and guidelines from the Center of Excellence in Genomic Medicine Research, Jeddah. Informed written consent was obtained from all the participants according to the Declaration of Helsinki. The study was also approved by the ethical committee of the Center of Excellence in Genomic Medicine Research, King Abdulaziz University. DNA was extracted from peripheral blood samples using the MegNA Pure 24 system following by the protocol (Roche life science, Penzberg, Germany). Pedigree was drawn by using information from the family. The DNA concentration was checked, and samples were prepared for WES according to the Agilent Sure Select Target Enrichment Kit preparation guide (Santa Clara, CA, United States). The blood samples were collected from all available members of the family and 100 unrelated healthy people of Saudi origin as controls as shown in Figure 1.

### Whole Exome Sequencing

To identify the basic pathogenic variant leading to disease phenotype, we performed WES using the Illumina HiSeq 2000/2500 system (San Diego, CA, United States). We set up our sampling by following the Agilent SureSelect Target Enrichment Kit preparation guide (Capture Kit, SureSelect v.6, Santa Clara, CA, United States). The libraries were sequenced utilizing the Illumina HiSeq 2000/2500 system. Diverse bioinformatics investigations were made to distinguish causative variant co-segregating for *BRPF1* phenotypes in an autosomal dominant manner. Whole exome sequencing data generate the raw reads in the form of FASTQ format. Insertion, deletion, and copy number variation were distinguished by utilizing SAMtools^1^. To use the BWA Aligner^2^, the crude information FASTQ files were adjusted. The resulting VCF file contains 111,850 variants. The variants were clarified by using different parameters, such as quality, frequency, genomic position, protein effect, and pathogenicity. The resulting sequences were compared against the hg19 human reference arrangement (National Center for Biotechnology Information assembly GRCh37)^3^. Furthermore, the obtained data were mapped against the information in the dbSNP^4^, population-based database such as genome aggregation database genomAD, Exome Variant Servise (ESP) and 1000 Genomes databases^5^ and disease-based database such as ClinVar (database of assertions about the clinical significance and phenotype relationship of human variations), OMIM (database of human genes and genetic conditions that also contains a representative sampling of disease-associated genetic variants), and Human Gene Mutation (Database of variant annotations published in the literature). The American College of Medical Genetics and Genomics and the Association for Molecular Pathology (ACMG). The variant detected here was classified as variant with unclear significance according to the most recent ACMG guidelines. *In silico* functional study was done for the current mutations to check deleterious effect and abnormalities caused by mutations. For the *in silico* predictions, we used software such as Mutation Tester^6^, 1000 Genomes^7^, PhyloP^8^, PhyloP GERP++^9^, SIFT^10^, PhastCons^11^, CADD^12^, SiPhy^13^, and Exome Aggregation Consortium^14^, and PROVEAN and MAPP for protein structure/function and evolutionary conservation. All the software predict this variant as disease causing.

### Sanger Sequencing

Whole exome sequencing results were validated using Sanger sequencing analysis. As a result, data files were obtained in the AB1 sequence trace format. Each sequence trace file was aligned to the corresponding reference sequence using the BioEdit and FinchTV software packages. We searched for the observed variations in the National Center for Biotechnology Information SNP database. To validate the position of the variant, primers were designed to flank and amplify the c.1054G > C in exon 3 of the *BRPF1* gene. *BRPF1*_forward, 5’-ACAGCAATGTCATCCTCTTCTGTG-3’; *BRPF1*_reverse, 5-ATGTCACATGGAAAGCTGTGTA-3. Further, to rule out this mutation in normal population, we also sequenced this variant in 100 control people.

### Computational Structural Analysis of the Mutant

The crystallographic model of the BRPF1 PZP domain was retrieved from the PDB (PDB accession no. 5ERC). The mutation was manually evaluated using the Pymol program (pymol.org).

## Data Availability Statement

The datasets for this article are not publicly available because family consents to share data publicly was not allowed. Requests to access the datasets should be directed to mimrannaseer@yahoo.com.

## Ethics Statement

The studies involving human participants were reviewed and approved by Center of Excellence in Genomic Medicine Research Center, King Abdulaziz University Jeddah, Saudi Arabia. Written informed consent to participate in this study was provided by the participants’ legal guardian/next of kin. Written informed consent was obtained from the individual(s), and minor(s)’ legal guardian/next of kin, for the publication of any potentially identifiable images or data included in this article.

## Author Contributions

MN, PP, and AC designed the experiments. AA, MA, and MN conducted the experiments. MA, PP, MN, and PP analyzed the data. SA and FG-V predicted the structural effect of the BRPF1 mutation. MN and AC wrote the manuscript. MN, MA, and PP proposed the research idea. All authors contributed to the editing of the manuscript and the scientific discussions.

## Conflict of Interest

The authors declare that the research was conducted in the absence of any commercial or financial relationships that could be construed as a potential conflict of interest.

## Footnotes

1 http://samtools.Sourceforge.Net/

2 http://bio-bwa.Sourceforge.Net/

3 http://genome.Ucsc.Edu/

4 http://www.Ncbi.Nlm.Nih.Gov/snp/

5 http://www.1000genomes.Org/data

6 http://www.mutationtaster.org/

7 http://www.internationalgenome.org/

8 https://www.ncbi.nlm.nih.gov/pmc/articles/PMC4702902/

9 http://mendel.stanford.edu/SidowLab/downloads/gerp/

10 http://sift.bii.a-star.edu.sg/

11 http://compgen.cshl.edu/phast/

12 https://cadd.gs.washington.edu/

13 https://omictools.com/siphy-tool

14 http://exac.broadinstitute.org/
